# A systematic study on the prevention and treatment of retinopathy of prematurity in China

**DOI:** 10.1186/s12886-018-0708-3

**Published:** 2018-02-14

**Authors:** Shuman Xu, Zhijiang Liang, Qiyun Du, Zhankui Li, Guangming Tan, Chuan Nie, Yang Yang, Xuzai Lv, Chunyi Zhang, Xianqiong Luo

**Affiliations:** 1 Guangdong Women and Children Hospital, 521 Xingnan Road, Guangzhou, Guangdong 511442 People’s Republic of China; 2 Hunan Women and Children Hospital, Changsha, Hunan People’s Republic of China; 3 Shaanxi Women and Children Hospital, Xi’an, Shaanxi People’s Republic of China

**Keywords:** Retinopathy of prematurity, Prevention and treatment, Medical institution, Investigation

## Abstract

**Background:**

To identify the prevention situation, the main factors influencing prevention effects and to develop control measures over retinopathy of prematurity in China.

**Methods:**

Using stratified random sampling method, we randomly selected 23 provincial and ministerial hospitals (8 in Guangdong province, 5 in Hunan province and 10 in Shaanxi province), 81 municipal hospitals (38 in Guangdong province, 19 in Hunan province and 24 in Shaanxi province), 180 district and county hospitals (76 in Guangdong province, 57 in Hunan province and 47 in Shaanxi province) in China. A total of 284 hospitals were enrolled in the study, with questionnaires distributed investigating the status and constrain factors of ROP presentation. Significant outcomes were analyzed thereafter by SPSS 19.0.

**Results:**

The screening rate of ROP in medical institutions from eastern, central and western China were 84.6%, 35.0% and 56.7%, respectively. The screening rate of tertiary and secondary medical institutions were 84.6% and 25.7% in the eastern, 35.0% and 4.9% in the central, 56.7% and 5.9% in the western region. Screening was carried out better in the tertiary than that in the secondary and primary institutions. Treatment for ROP was available in 15.7% of all the tertiary hospitals surveyed. Lack of professionals, equipments and technologies were considered to be major restrain factors for screening.

**Conclusions:**

The ROP screening and treatment status have demonstrated significant regional diversity due to uneven distribution of medical resources in China. Developed areas had established intraregional cooperation models, whereas less-developed areas should consider set up a large-scale, three-level ROP prevention network. It is of paramount importance that education and training towards ophthalmologists should be vigorously strengthened. It is strongly recommended that implement ROP telemedicine and integrated ROP prevention and management platforms through the Internet should be established.

**Electronic supplementary material:**

The online version of this article (10.1186/s12886-018-0708-3) contains supplementary material, which is available to authorized users.

## Background

Retinopathy of prematurity (ROP) is one of the common complications that develops in infants born prematurely, which can eventually lead to retinal detachment and blindness in severe cases. Clinical research shows that the lower birth weight and smaller the gestational age is, the higher incidence of ROP appears, indicating an inverse relationship between the occurrence of ROP and the birth weight, along with gestational age [1, 2]. Although the mortality of premature infants has dropped considerably around the world owing to continuous improvement of health management in NICU [3], morbidity of ROP remains rising in past 10 years in China. ROP has been assumed to be the leading cause of childhood blindness by the ‘Vision 2020 project’ of WHO [4]. It is estimated that about five million children with ROP lost their vision permanently worldwide each year, of which 4000 children come from China, accounting for 1.9% of the total blinding rate in children under five [5]. Studies on the blind schools of Guangzhou [6] and Shanghai [7] have both described that ROP ranks the No.1 cause of blindness, with the proportion of 32.88% and 18.2% in the two places, respectively. Meanwhile, untimely treatment can also result in children blindness. Standard screening and timely treatment of ROP are therefore urgently needed to prevent premature infants from being blind. This study aimed to investigate the status, existing problems and critical factors of prevention and treatment for ROP in premature infants in China, and ultimately, to explore suitable counter measures.

## Methods

Two hundred eighty-four medical institutions obtained by sampling were studied as our research subjects, with the inclusion criterion of hospitals setting up with Neonatal Intensive Care Unit (NICU) or eye hospitals providing with ROP services.

### Sampling method

We employed stratified random sampling for this study. We randomly chose three provinces based on the location and level of economic development. These provinces were Guangdong province in the eastern region, Hunan province in the central region and Shaanxi province in the western region. Considering the representativeness of hospitals and the feasibility of manpower and material resources, we then randomly selected 20% from the hospitals at three levels which met the inclusion criterion in each province. These hospitals were 23 provincial and ministerial hospitals (8 in Guangdong province, 5 in Hunan province and 10 in Shaanxi province), 81 municipal hospitals (38 in Guangdong province, 19 in Hunan province and 24 in Shaanxi province), 180 district and county hospitals (76 in Guangdong province, 57 in Hunan province and 47 in Shaanxi province), amounting to 284 hospitals overall.

### Main outcomes

The hospital grade and administrative level of survey hospitals, the screening and treatment situation of the hospitals, restraining factors of ROP screening, such as personnel and funding shortage, space limitation, and lack of concern.

### Data collection and quality control

Data were collected in form of questionnaires (Additional files 1 and 2). Provincial women and children hospitals, regarding as our research center, were responsible for arranging the whole research progress and connecting with other hospitals by signing cooperation agreements, in this way, to ensure high recovery rate. We assigned one investigator to take charge of the questionnaire distribution and recovery. Questionnaires were distributed to the director of neonatology department who undertakes the responsibility of both the ROP prevention and department management in each hospital. The reliability and validity of the questionnaire was tested and Cronbach’s α was 0.962. A total of 284 questionnaires were distributed and all were recovered with a recovery rate of 100%.

### Data analysis

All quantitative data were entered into Epidata 3.1 twice to ensure accuracy after careful examination. Meanwhile, missing or outliers were timely corrected through logic correction. We mainly conducted descriptive statistics methods and chi-square test for the data analysis using SPSS (version 19.0).

Our research is a status analysis, without involving any human or animal data, and approved by the Medical Ethics Committee of Guangdong Women and Children Hospital. All the surveyed hospitals had been informed before the investigation and consented to be participants.

## Results

The screening rate of ROP reached the highest (84.6%) in the tertiary hospitals of eastern area, much higher than that of central (35.0%) or western (56.7%) hospitals. The difference between the areas was statistically significant (*p* < 0.05). The screening rate in secondary and primary hospitals was 25.7% in the eastern area, higher than those of central (4.9%) or western (5.9%) area. In general, ROP screening was carried out better in the tertiary hospitals than those at lower levels. Compared to the central and western areas, the proportion of hospitals which carried out ROP screening is higher in the eastern region (*p* < 0.05), as shown in Table 1.

**Table 1:** The ROP screening rates of medical institutions from eastern, central and western China

Survey Content	Tertiary medical institutions			Secondary and primary medical institutions		
	Eastern region (52)	Central region (20)	Western region (30)	Eastern region (70)	Central region (61)	Western region (51)
ROP screening	44/84.6%	7/35.0%	17/56.7%	18/25.7%	3/4.9%	3/5.9%

Tertiary medical institutions compare: χ2 = 17.91 *P* = 0.000

Secondary and below medical institutions compare: χ2 = 10.57 *P* = 0.005

Table 2 indicates that in all the 284 hospitals under investigation, only 16 hospitals are capable to carry out laser treatment for ROP, which including 12 tertiary hospitals (accounting for 15.7% in all the tertiary hospitals) and 4 secondary and primary hospitals (accounting for 2.2% in all the secondary and primary hospitals), demonstrating that the service of laser treatment is gravely insufficient cross China.

**Table 2:** The number of medical institutions to carry out ROP laser treatment(N)

ROP Treatment	Tertiary medical institutions			Secondary and primary medical institutions		
	Eastern region	Central region	Western region	Eastern region	Central region	Western region
Laser therapy	8	1	3	4	0	0
Condensation therapy	3	1	2	1	0	0

Table 3 indicates that about 80% of the ROP screening was conducted by tertiary hospitals, while 12.9% to 20.3% of the screening was performed in secondary and primary hospitals (χ2 = 499.5, *p* = 0.000). The reason for not carrying out ROP screening varies between hospitals of different areas, as summarized in Table 4. First and foremost, absence of professionals is the leading cause, with the high proportion of 75% to 84.6% in the tertiary hospitals and 85.7% to 94.1% in the secondary and primary hospitals. Health care resources shortage is likely to be the second reason, of which the proportion ranges from 23.1% to 61.5% in the tertiary hospitals and 31.9% to 41.9% in the secondary and primary hospitals. Holding the view of thinking referral more safety ranks third of all the possible causes, covering 23.1% to 62.5% in the tertiary hospitals and 29.4% to 39.5% in the secondary and primary hospitals. Lack of sites becomes the fourth reason, showing the rate of 15.4% to 37.5% in the tertiary hospitals and 20.8% to 47.1% in the secondary and primary hospitals. No significant difference was discovered between the regions (*p* > 0.05) in terms of the above four reasons. In addition, Insufficient input and output was demonstrated by some hospitals as a potential cause, accounting for 23.1% to 37.5% in the tertiary hospitals and 9.8% to 10.4% in the secondary and primary hospitals in part of the areas.

**Table 3:** The ROP screening number from 2010 to 2012(n/%)

Organization level	2012	2011	2010
Tertiary medical institutions	30,547/87.1%	23,045/83.3%	16,125/79.7%
Secondary and below medical institutions	7799/12.9%	4630/16.7%	2382/20.3%
Sum	38,346	27,675	18,507

χ2 *=* 499.5 *P* < 0.0001

**Table 4:** Reasons for not carrying out the ROP screening in the hospitals

Reasons for not to carry out the ROP screening	Hospital num	Tertiary medical institutions			Secondary and below medical institutions		
		Eastern region	Central region	Western region	Eastern region	Central region	Western region
Hospital Sum	176	8	13	13	48	51	43
Missing hospitals	16	0	0	0	4	7	5
Personnel shortage^1^	158	6/75.0%	10/76.9%	12/84.6%	42/85.7%	48/94.1%	38/88.4%
Capital shortage^2^	71	4/50.0%	3/23.1%	8/61.5%	15/31.9%	23/45.1%	18/41.9%
Consider referral safer^6^	61	5/62.5%	3/23.1%	5/38.5%	16/33.3%	15/29.4%	17/39.5%
Space limitation^3^	51	3/37.5%	2/15.4%	2/15.4%	10/20.8%	24/47.1%	10/23.3%
Lack of concern^4^	27	0/0	1/7.7%	3/23.1%	10/20.8%	8/15.7%	5/11.6%
Insufficient input and output^7^	16	3/37.5%	0/0	3/23.1%	5/10.4%	5/9.8%	0/0
Consider unnecessary^5^	5	0/0	0/0	2/15.4%	2/4.2%	1/2.0%	0/0
Others^8^	18	1/12.5%	5/38.5%	3/23.1%	4/8.3%	1/2.0%	4/9.3%

^1^Tertiary medical institutions compare: χ2 *=* 0.360 *P* = 0.835

Secondary and below medical institutions compare: χ2 *=* 1.956 *P* = 0.376

^2^Tertiary medical institutions compare: χ2 *=* 4.047 *P* = 0.132

Secondary and below medical institutions compare: χ2 *=* 1.894 *P* = 0.388

^3^Tertiary medical institutions compare: χ2 *=* 1.830 *P* = 0.401

Secondary and below medical institutions compare: χ2 *=* 0.736 *P* = 0.698

^4^Tertiary medical institutions compare: χ2 *=* 23.16 *P* = 0.000

Secondary and below medical institutions compare: χ2 *=* 2.877 *P* = 0.237

^5^Tertiary medical institutions compare: χ2 *=* 3.433 *P* = 0.180

Secondary and below medical institutions compare: χ2 *=* 0.756 *P* = 0.685

^6^Tertiary medical institutions compare: χ2 *=* 3.260 *P* = 0.196

Secondary and below medical institutions compare: χ2 *= 0*.489 *P* = 0.783

^7^Tertiary medical institutions compare: χ2 *=* 5.219 *P* = 0.074

Secondary and below medical institutions compare: χ2 *=* 0.902 *P* = 0.637

^8^Tertiary medical institutions compare: χ2 *=* 1.839 *P* = 0.399

Secondary and below medical institutions compare: χ2 *=* 17.69 *P* = 0.000

No funds means there are no funds are used to purchase ROP screening equipment

The incidence of ROP has indicated to be closely related to the treatment level of the facilities. Hospitals having neonatology department and neonatal intensive care center (NICU) sums to 207 (84.1%) and 155 (63.0%), respectively, of all the 284 sample hospitals in our study. However, the medical resource allocation of ROP prevention and treatment has turned up to be unevenly distributed in the hospitals of different regions and levels. With most of the resources assembled in the eastern area, neonatology department and NICU were set up in all the tertiary hospitals and largely set up in the secondary and primary hospitals (64 hospitals having neonatology department and 47 hospitals having NICU department). On the contrary, neonatology and NICU department can hardly be found in the secondary and primary hospitals (only 9 hospitals having neonatology department and 6 hospitals having NICU department) in the western area.

## Discussion

### The ROP screening and treatment in China has demonstrated obvious regional specificity due to the uneven distribution of resources. Various regions can establish suitable ROP prevention models

The serious imbalance of economic development and resource allocation across China have led to the disproportion of ROP screening. There is a lack of a universally applicable, reasonable and sound ROP screening system, which started relatively late in most regions. Our study showed that ROP screening and treatment were carried out more intensively in the tertiary hospitals than that of secondary and primary hospitals in all regions. The majority of ROP screening work was conducted in the tertiary hospitals which were equipped with adequate resources. Our finding was in line with the investigation on 109 hospitals across China by Feng Zhichun [8], indicating that tertiary hospitals did better than other hospitals with a screening rate of 73.78% compared to 25%. We also found obvious regional diversity of ROP prevention due to the higher level of economic development and much more abundant resource allocation in the eastern region than the central and western regions. In general, medical institutions which are capable to carry out ROP screening were allocated unevenly and were relatively a small amount all over China. However, decrease of severe ROP incidence reported in recent years demonstrated that the ROP screening and treatment status was basically improving.

Collaborations between institutions has proved to be one of the key point that need attention in ROP detection and treatment programs [9]. At present, some developed regions in China, such as Beijing and Shanghai, have already carried out intraregional cooperation model, which has greatly improved the screening coverage and early treatment of ROP. For instance, the ‘Eye Center Model’ in Beijing which is carried out by an ophthalmic centre and in alliance with several NICUs, has achieved a high screening rate of 87.3% [10]. The rate was reported to be 85.26% in the co-established ROP screening cooperation net by 13 hospitals in Eastern China [11]. ‘Inter-hospital Cooperation Model’ was built up in Shenzhen Eye Hospital and other hospitals by signing agreements, creating the green channel of ROP screening and consultation to ensure timely referral, treatment and mutual benefit. In recent 10 years, the incidence of severe ROP cases in Shenzhen has decreased significantly [12, 13]. All the above regions have strong sophistication and superiority in both facilities and technology. They can not only undertake local ROP screening work but also can conduct the treatment of severe referral ROP patients elsewhere. Therefore, conditional eastern regions can draw on such models and establish cooperation model of ROP prevention within the region to jointly carry out ROP screening, consultation, treatment and follow-up.

As for the central and western regions with relatively lower level of economic development, establishing a large-scale and comprehensive ROP prevention network is likely to be a more appropriate and feasible way, such as the construction practices of three-level ROP prevention network in Shaanxi province [14]. It is suggested that one or several tertiary hospitals located in relatively developed cities, which possess adequate professional medical equipment and professional technical teams, can be established as the ROP prevention center of the whole region, while municipal and district hospitals were as the secondary units of the network, county and township hospitals were as the primary units. Meanwhile, admittance and cooperation systems for ROP prevention institutions should be formulated to clarify standard ROP screening, especially for the primary units. The guiding role of the prevention center should be fully emphasized on realizing resource sharing, establishing complete ROP prevention and management systems, reducing the incidence and disability rate of ROP and gradually improving the accessibility of ROP screening and treatment in central and western China.

### Personnel shortage becomes the main restriction of ROP prevention. Education and training should be vigorously strengthened

A survey in 2006 has found that only half of the specialists were willing to take on ROP-related work in the US and lack of human resources was the main problem of ROP screening in western developed countries [15]. Confronted with ophthalmologist shortage in the nations around the world, it is therefore imperative to strengthen the education training of ophthalmologists and relevant professionals. Our study also revealed that lack of clinical professionals was the main barrier of ROP screening, since the number of doctors engaged in screening was far behind demands. Awareness of ROP prevention of department directors and hospital administrators for primary hospitals should be raised, so as to further promote the screening smoothly. Early examination should be done to the premature infants by professionals with systematic knowledge of ROP screening and treatment to ensure safety, efficiency and accuracy. Faced with adult patients and complicated ophthalmic examination, the enthusiasm of ophthalmologists can hardly be motivated in most general hospitals. Consequently, it is quite necessary to establish relevant incentive systems to motivate doctors. Detailed cultivating schemes for professionals of ROP prevention should be formulated. Theory and skill training of ROP screening and treatment-related field, including prevention and treatment of preterm birth, respiratory support technology, oxygen therapy management, new recovery technology should be regularly held through multiple forms such as courses, seminars and on-site technical guidance to promote the capacities of doctors at the frontline.

### Information technology should be fully used to promote ROP telemedicine, so as to explore building integrated management platforms for ROP prevention

With the rapid development of science and technology, telemedicine emerges and brings new ideas to ROP prevention. Compared to traditional face-to-face medical, telemedicine is of higher efficiency and can beyond geographical limitations and improve the availability and timeliness of medical service for patients in remote areas. Moreover, the popularization and easy operating of RetCam makes ROP telemedicine more accessible [16]. Images can be captured through digital imaging techniques by technicians and sent to the ROP specialists for timely disease detection. Fijalkowski also claimed that telemedicine has high accuracy for detecting clinically significant ROP in time [17]. Currently, telemedicine has already been applied in some developed regions in eastern China and is necessary to further generalized in the central and western regions. For the present, the ‘Internet+’ model has been extensively carried out in China while some medical institutions begin to develop ‘Cloud Medical Treatment’ and ‘Wisdom Hospital’. This undoubtedly provides a favorable condition for ROP prevention. Where possible, establishment of integrated, interactive prevention and management platform including ROP screening, treatment, consultation and follow-up function through the Internet and open up to the medical institutions and personnel to share ROP prevention information and case resources, timely update forefront knowledge and online discussion should be taken into account. Simultaneously, the introduction and development of ‘mobile medical treatment’ also provides great convenience to medical providers. The combination of ROP prevention and ‘mobile medical treatment’ should be highlighted in the future.

## Conclusions

In conclusion, the ROP screening and treatment have demonstrated obvious regional specificity with better implementation in the eastern region due to the uneven distribution of medical resources in China. Intraregional cooperation model of ROP prevention has been established in eastern China. Less-developed areas can explore to set up a large-scale, three-level ROP prevention network to share medical resources. Cope with the personnel shortage, it is of paramount importance that education and training should be vigorously strengthened towards ophthalmologists. It is strongly recommended to implement ROP telemedicine and establish integrated ROP prevention and management platforms through the Internet so as to maximize the benefits of ROP prevention within limited resources.

### Limitations

In this study, we mainly used survey to investigate the constrain factors of ROP screening. So there may exists some reporting bias because it depends heavily on self-voluntary response.

## Additional files

Additional file 1: Investigation on the prevention situation and influencing factors of ROP for medical institutions. (PDF 105 kb)

Additional file 2: Investigation on the ROP prevention knowledge, attitude and behavior for medical staff. (PDF 38 kb)

## Abbreviation

ROP: Retinopathy of prematurity

## References

[1] Ping F, Peiquan Z (2013). Research status and progress of retinopathy of premature infants- summary of the third world congress of preterm infants[J]. Chinese journal of ocular fundus Diseases.

[2] Borroni C (2013). Survey on retinopathy of prematurity (ROP) in Italy[J]. Ital J Pediatr.

[3] Liu L, Johnson HL, Cousens S (2012). Global, regional, and national causes of child mortality: an updated systematic analysis for 2010 with time trends since 2000[J]. Lancet.

[4] Gilbert C, Foster A (2001). Childhood blindness in the context of VISION 2020—the right to sight[J]. Bull World Health Organ.

[5] Gilbert C (2008). Retinopathy of prematurity: a global perspective of the epidemics, population of babies at risk and implications for control[J]. Early Hum Dev.

[6] Yingfeng Z, Binhuang L, Yong W, Mingguang H (2007). An investigation on the causes of blindness and low vision of students in blind School in Guangzhou[J]. Eye science.

[7] Guifang J, Hongmei X, Hongfen S. The analysis of shanghai city survey of etiology and vision of blind students [J]. Recent advances in Ophthalmology. 2006;26:622-3.

[8] The Coordination Group for Present Situation of Neonatal Subspecialty in the Mainland of China (2013). Present situation of retinopathy of prematurity management in mainland China: a survey based on 109 hospitals[J]. Chin J Appl Clin Pediatr.

[9] Quinn GE (2016). Retinopathy of prematurity blindness worldwide: phenotypes in the third epidemic[J]. Eye and Brain.

[10] The Investigation Group of Retinopathy of Premature Infants in Beijing (2008). Analysis of screening and risk factors of retinopathy of premature infants in Beijing[J]. Chinese journal of ophthalopathy.

[11] Xiaoxin N (2004). The characteristics and screening guide of retinopathy of premature infants in China[J]. Chinese journal of ophthalopathy.

[12] Zhaojie C, Yusheng W (2012). The incidence of retinopathy of premature infants in the mainland of China in recent 20 years[J]. Chinese. J Ophthalmol.

[13] Retinopathy of premature infants in Shenzhen. Analysis of the incidence of retinopathy of premature infants in Shenzhen area in 10 years[J]. Chinese Journal of Ocular Fundus Diseases 2014; 30: 12–16.

[14] Wang Y (2014). Ideas and practices of construction of tertiary network of retinopathy of prematurity in China[J]. Chinese journal of ophthalopathy.

[15] Chan RVP, Williams SL, Yonekawa Y, et al. Accuracy of retinopathy of prematurity diagnosis by retinal fellows. Retina. 2010;30:958-965. 10.1097/IAE.0b013e3181c9696a PMC2884082 20168274

[16] Weaver DT (2013). Telemedicine for retinopathy of prematurity[J]. Curr Opin Ophthalmol.

[17] Fijalkowski N, Zheng LL, Henderson MT, Wallenstein MB, Leng T, Moshfeghi DM (2013). Stanford university network for diagnosis of retinopathy of prematurity: four-years of screening with telemedicine[J]. Curr Eye Res.

