# Supplementary material for: A systematic study on the prevention and treatment of retinopathy of prematurity in China
Source: BMC Ophthalmol. 2018 Feb 14;18:44. doi: 10.1186/s12886-018-0708-3 (PMC5813392; doi:10.1186/s12886-018-0708-3)
Supplement: Supplementary file 1 — Investigation on the prevention situation and influencing factors of ROP for medical institutions. (PDF 105 kb) [file 12886_2018_708_MOESM1_ESM.pdf]

Number □□□□-□□

(Municipal and county administrative division code-institution serial number)

Investigation on the prevention situation and influencing factors of ROP for medical institutions

Investigation area: ___________City___________District (County)

Name of investigation institution: ____

Signature_____________; **Contact number**________________; **Date**___________________

1. General information of hospital

1.1 Hospital class:

□ primary hospital □ secondary hospital □ tertiary hospital □ undecided

1.2 Hospital grade:

□ special-grade hospital □ grade A □ grade B □ undecided

1.3 Administrative level of hospital:

□ ministerial and provincial-level □ prefecture-level □ district-level □ county-level

2. ROP prevention situation

Status of Neonatology Department and Neonatal Intensive Care Unit (NICU):

Established time of Neonatology Department___________, Established time of NICU______

2.1 The number of beds and service (Filling with numbers of year 2012)

Hospitalized patients of Neonatology Department: _________cases

Total number of beds of Neonatology Department: _________beds, including number of beds of NICU_________beds

Total number of beds of Obstetrics Department:__________beds, annual delivery number___

ROP related service in 2012:

Annual receive and cure number of premature babies: ________cases

< 2500g (low-birth weight infant, LBW): ________cases

< 2000g (including very/extremely low birth weight infant): ________cases

< 1500g (very low birth weight infant, VLBW): _________cases

< 1000g (extremely low birth weight infant, ELBW): _________cases

2.2 Personnel and training (Filling with numbers of year 2012)

Numbers of doctors engaged in ROP screening: ______________(if 'no', fill in 0)

ROP related training:

Neonatal physician_______person-time/year; Ophthalmologist_________person-time/year

Hospital administrators_________person-time/year

2.3 Gross area of Neonatology Department (Filling with numbers of year 2012)

Total area_________㎡, including NICU__________㎡

2.4 Specialized apparatuses of Neonatology Department (If 'yes', fill in the counts)

Negative pressure aspirator: □ yes □ no phototherapy box: □ yes □ no

induction equipment (non-contact): □ yes □ no mask: □ yes □ no

neonatal rescue platform: □ yes □ no incubator: □ yes □ no

recovery capsule: □yes □no infusion pump: □yes □no

island of life: □yes □no bedside X-ray machine: □yes □no

transit cases: □yes □no high frequency ventilator: □yes □no

conventional mechanical ventilator: □yes □no nasal CPAP ventilator: □yes □no

monitor: □yes □no hypothermia therapy apparatus: □yes □no

Nitric oxide therapy apparatus: □yes □no transport ventilators ： □yes □no

infant hyperbaric oxygen chamber: □yes □no laryngoscope: □yes □no

glucometer: □yes □no

T-piece resuscitator (accorded with 2011 recovery guidelines requirements): □yes □no

2.5 ROP related equipment (If 'yes', fill in the counts)

oxygen-air mixer: □yes □no oxygen concentration meter: □yes □no

blood-gas analyzer: □yes □no

oxygen saturation monitor (Any containing oxygen saturation determination): □ yes □ no

direct ophthalmoscopy: □yes □no indirect ophthalmoscopy: □yes □no

fundus digital camera: □yes □no condensing therapy apparatus : □yes □no

RatCam Ⅲ: □yes □no fundus laser treatment instrument: □yes □no

Other diagnostic equipment for ROP____

Newborn resuscitation equipment in the delivery room and operating room:

oxygen-air mixer: □yes □no compressed air: □yes □no

T-piece resuscitator (accorded with 2011 recovery guidelines requirements): □yes □no

nosocomial transhipment: transport incubator: □yes □no

Emergency transport equipment (Fill in when hospital carries out newborn transshipment)

compressed air: □yes □no compressed oxygen: □yes □no

transport ventilators ： □yes □no transport incubator: □yes □no

infusion pump: □yes □no vehicle-mounted power interface : □yes □no

vacuum device: □yes □no monitor: □yes □no

oxygen-air mixer: □yes □no

T-piece resuscitator (accorded with 2011 recovery guidelines requirements): □ yes □ no

2.6 Technical projects undertaken

fiberoptic bronchoscopy: □yes □no cardiopulmonary monitoring: □yes □no

bedside blood gas analysis: □yes □no double change: □yes □no

bedside cardiac color ultrasound: □ yes □ no bedside radiography: □ yes □ no

spiral CT vascular airway reconstruction: □ yes □ no MRI: □ yes □ no

mild hypothermia therapy: □ yes □ no ROP screening and treatment: □ yes □ no

umbilical arteriovenous catheterization: □ yes □ no PICC: □ yes □ no

invasive artery blood pressure monitoring: □ yes □ no CRRT: □ yes □ no

peritoneal dialysis: □ yes □ no renal biopsy: □ yes □ no

esophageal PH value determination: □ yes □ no NO inhalation therapy: □ yes □ no

PS replacement therapy: □ yes □ no mechanical ventilation: □ yes □ no

high freguency oscillatory ventilation: □ yes □ no

noninvasive nasal congestion assisted ventilation: □ yes □ no

high flow oxygen therapy: □ yes □ no

closed thoracic drainage: □ yes □ no neonatal screening: □ yes □ no

neonatal surgery: □ yes □ no pediatric cardiothoracic surgery: □ yes □ no

2.7 ROP prevention situation：

2.7.1 Conducting oxygen therapy monitoring: □ yes □ no

2.7.2 Conducting ROP screening: □ yes □ no

Reasons for not carrying out ROP screening:

□ Personnel shortage □ Funding shortage

□ Space limitation □ Lack of concern

□ Consider unnecessary □ Consider referral safer

□ Insufficient input and output □ Others_____________

Time for carrying out ROP screening: ______________

Forms of screening cooperation: □ conducted by doctors inside the hospital □ engaging experts outside the hospital □ referral to other hospital

Total number of ROP screening cases in 2010________, positive cases_________

Positive cases over □ period_____, including □ period cases_____, □ period cases_____

Total number of ROP screening cases in 2011________, positive cases________

Positive cases over □ period_____, including □ period cases_____, □ period cases_____

Total number of ROP screening cases in 2012________, positive cases________

Positive cases over □ period_____, including □ period cases_____, □ period cases_____

2.7.3 ROP related referral and transshipment situation in the absence of ROP screening conditions:

□ referral at birth □ referral when ROP is suspected □ no referral at any time

2.7.4 ROP treatment referral situation in the absence of ROP treatment conditions:

□ immediate referral when ROP is screened □ referral when ROP threshold is screened

□ no referral □ main institution of referral____________

2.7.5 Conducting ROP laser treatment in 2012: □ yes □ no

treatment cases_______ ROP dispute cases_______ ROP disputes (time, reason)________

2.7.6 Conducting ROP condensate treatment in 2012: □ yes □ no

treatment cases:_______ cases of IV period ROP_______ cases of V period ROP______

ROP treatment way：□ separate □ cooperative □ others

cooperative hospital and its level___________________, cooperative way________________

2.7.7 Will the exact time and place of ROP screening be informed when the premature was discharged from the hospital without screening or discharged automatically: □ yes □ no

2.7.8 ROP screening mode (Please choose suitable mode for your hospital and give a brief explanation)

Mode 1: □ In cooperation with other hospitals to carry out ROP screening

Mode 2: □ Conducted by trained ophthalmologists inside the hospital

Mode 3: □ Executing diagnosis by ophthalmologist after fundus examination conducted by neonatal physician

Reason 1: □ No condition for ROP screening inside the hospital

Reason 2: □ Resource sharing

Reason 3: □ Ensure screening quality, including timeliness, accuracy and screening rate

Reason 4: □ Others____

3. A feasibility investigation on the prevention and management mode of retinopathy of premature

The prevention and management mode of ROP, as one of the previous research results of our team, was designed based on the system management theory, as shown in the figure below. Please give brief advice on the existing problems and suggestions of the mode promotion in local area in combination with your own reality.

ROP prevention and management mode is as follows:

Health administrative agency and the provincial management center can formulate policies, system and goals, give support on the equipment and funding, cultivate ROP prevention professionals, and conduct quality evaluation and supervision on the regional ROP prevention institutions according to the reported information by network throughout the province.

Regional ROP prevention institutions, relied on the tertiary hospitals, can establish sound patient referral and consultation system and give guidance to the local hospitals for better ROP prevention. Department of neonatology and ophthalmology, in the context of a good cooperation, should work together on ROP screening, diagnosis, treatment and follow-up, while department of obstetrics and gynecology should make efforts for premature birth prevention, exhaustive pregnancy test and prenatal health education.

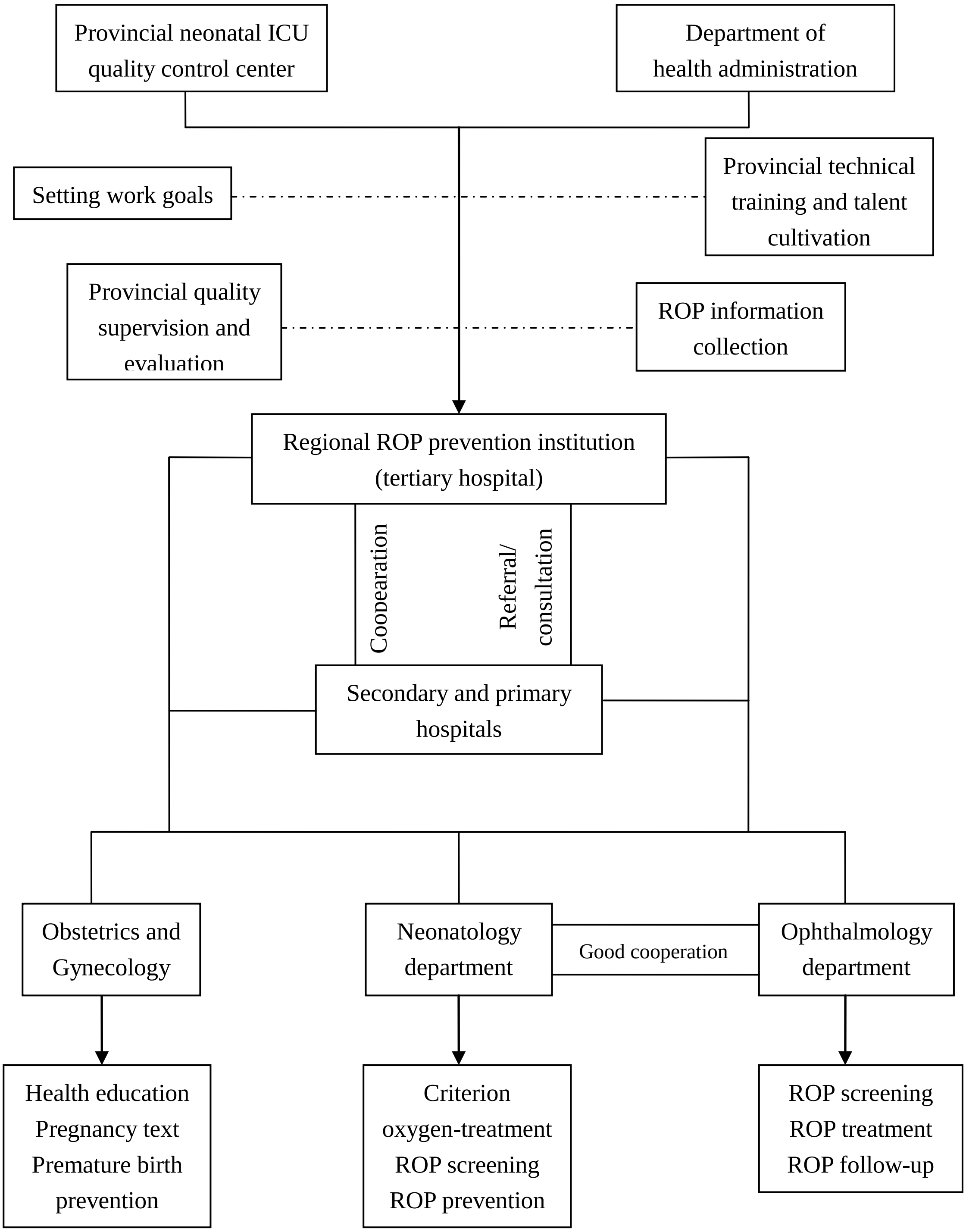

Recommendations on the ROP prevention and management mode:

1. What do you think of the operability of this mode ?

2. Do you think the mode is suitable for your own circumstances ?

3. Do you have any suggestions for revision on this mode ?

4. How many points will you give to this mode on a 100-point scale?

5. What do you think is the main barrier to the mode operation ?
