# Supplementary material for: A systematic study on the prevention and treatment of retinopathy of prematurity in China
Source: BMC Ophthalmol. 2018 Feb 14;18:44. doi: 10.1186/s12886-018-0708-3 (PMC5813392; doi:10.1186/s12886-018-0708-3)
Supplement: Supplementary file 2 — Investigation on the ROP prevention knowledge, attitude and behavior for medical staff. (PDF 38 kb) [file 12886_2018_708_MOESM2_ESM.pdf]

Investigation on the ROP prevention knowledge, attitude and behavior for medical staff

The incidence of retinopathy of prematurity (ROP) have been rapidly increased in recent ten years in China and become one of the main causes of premature disability. So it is of great urgent to carry out systematic ROP prevention.

We conduct this survey to understand your knowledge and skills about ROP and to collect advice for strengthening ROP prevention and services. All the information you provide will be kept secret and used only for statistical analysis. This survey is completely voluntary and thanks for your participation and cooperation.

1. What is the administrative level of your hospital ?

□ ministerial and provincial-level □ prefecture-level

□ district-level □ county-level

2. What is your job ?

□ nurse □ neonatal physician □ ophthalmologist

□ administrative staff □ others

3. Is there ROP prevention guidelines in China?

□ yes □ no □ I don't know

4. If you think there is ROP prevention guidelines, how do you know it?

□ data inquiry □ training course

□ department learning □ told by peer

5. What do you think of the implementation of the guidelines in your hospital ?

□ implemented well □ partly implemented □ not implemented

6. What is the reason for not implementing the guidelines ?

□ unnecessary □ no conditions

7. Have you ever got any ROP knowledge training ?

□ yes □ no

8. What kind of training have you taken ?

□ ROP lectures on continue education training □ thematic training for ROP □ attendance in advanced studies for ROP □ others

9. Do you think it is necessary for training ?

□ yes □ no

10. Supervision situation of ROP by health administration department:

□ yes (level of the health administration department_________) □ no

11. What do you think of the implementation of ROP screening in the medical institutions ?

□ very important □ important □ so-so

□ not important □ I don't care

12. Does your hospital carry out ROP screening ?

□ yes, it is carried out to the premature whose weight are below 2000g

□ yes, it is carried out to the newborn who have been treated with oxygen

□ no, never □ I don't know □ others

13. Have there any medical disputes related to ROP in your hospital ?

□ yes □ no □ I don't know

14. What do you think is the main limitation for not carrying out ROP screening in some medical institutions ?

□ lack of concern □ personnel shortage □ funding shortage

□ apparatus shortage □ unnecessary

15. How many years have your hospital implemented ROP screening ?

□< 1 year □1-2 years □2-3years

□>3 years □ not implemented

16. How many doctors who have the capability of ROP screening in your hospital ?

□0 □1 □2 □3 □≥4

17. What is the job of the doctors who conduct retinal screening ?

□ ophthalmologist □ neonatal physician □ physician of child healthcare

18. What is the source of the doctors who conduct ROP screening in your hospital ?

□ recruit as required □ training of existing medical staff

□ get support from other hospitals □ never conducted ROP screening

19. Do you think it is necessary for establishing regional ROP screening center ?

□ absolutely necessary □ necessary □ a little

□ not necessary □ I don't care

20. Which do you prefer to the function of the regional screening center ?

□ help train medical staff in the hospital

□ on-site professional guidance regularly

□ newborns transferred to the center to do screening

□ all the above

□ no need for a center

Place: ______City______District (County)

Institution: ______________________________

Date: ______________________________
